# 2-Phenyl­imidazo[1,2-*a*]pyridine-3-carbaldehyde

**DOI:** 10.1107/S1600536808011306

**Published:** 2008-04-26

**Authors:** Abderrahman Anaflous, Hanane Albay, Nour-eddine Benchat, Brahim El Bali, Michal Dušek, Karla Fejfarová

**Affiliations:** a Département de Chimie, Faculté des Sciences, BP 717, 60000 Oujda, Morocco; b Laboratory of Mineral Solid and Analytical Chemistry, ‘LMSAC’, Department of Chemistry, Faculty of Sciences, University Mohamed I, PO Box 717, 60000 Oujda, Morocco; c Institute of Physics, Na Slovance 2, 182 21 Praha 8, Czech Republic

## Abstract

In the title compound, C_14_H_10_N_2_O, the dihedral angle between the imidazo[1,2-*a*]pyridine and phenyl rings is 28.61 (4)° The mol­ecules are connected into broad chains parallel to the *a* axis by weak C—H⋯O and C—H⋯N hydrogen bonds. The linking of the ribbons is provided by π–π stacking inter­actions between neighbouring pyridine rings, with a centroid–centroid distance of 3.7187 (7) Å.

## Related literature

For general background, see Anaflous *et al.* (2008 ▶) and references therein. For related literature, see: Meth-Cohn & Stanforth (1991 ▶).
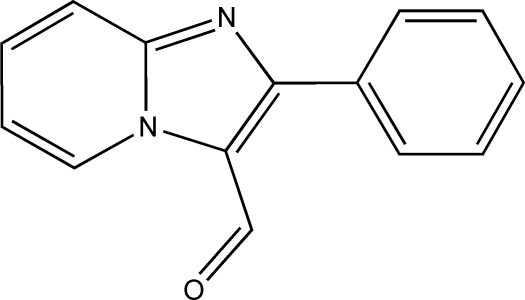


## Experimental

### 

#### Crystal data

C_14_H_10_N_2_O
*M* *_r_* = 222.2
Orthorhombic, 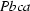

*a* = 13.0640 (3) Å
*b* = 7.4162 (2) Å
*c* = 21.6698 (6) Å
*V* = 2099.48 (9) Å^3^
*Z* = 8
Mo *K*α radiation
μ = 0.09 mm^−1^
*T* = 120 K
0.57 × 0.40 × 0.24 mm

#### Data collection

Oxford Diffraction Xcalibur2 diffractometer with Sapphire2 CCD detector
Absorption correction: none
25795 measured reflections
2196 independent reflections
1305 reflections with *I* > 3σ(*I*)
*R*_int_ = 0.049

#### Refinement

*R*[*F* ^2^ > 2σ(*F* ^2^)] = 0.031
*wR*(*F* ^2^) = 0.077
*S* = 1.04
2196 reflections
154 parameters
H-atom parameters constrained
Δρ_max_ = 0.15 e Å^−3^
Δρ_min_ = −0.16 e Å^−3^

### 

Data collection: *CrysAlis CCD* (Oxford Diffraction, 2006 ▶); cell refinement: *CrysAlis RED* (Oxford Diffraction, 2006 ▶); data reduction: *CrysAlis RED*; program(s) used to solve structure: *SIR2002* (Burla *et al.*, 2003 ▶); program(s) used to refine structure: *JANA2000* (Petříček *et al.*, 2000 ▶); molecular graphics: *DIAMOND* (Brandenburg & Putz, 2005 ▶); software used to prepare material for publication: *JANA2000*.

## Supplementary Material

Crystal structure: contains datablocks global, I. DOI: 10.1107/S1600536808011306/bg2180sup1.cif

Structure factors: contains datablocks I. DOI: 10.1107/S1600536808011306/bg2180Isup2.hkl

Additional supplementary materials:  crystallographic information; 3D view; checkCIF report

## Figures and Tables

**Table 1:** Hydrogen-bond geometry (Å, °)

*D*—H⋯*A*	*D*—H	H⋯*A*	*D*⋯*A*	*D*—H⋯*A*
C4—H4⋯N1^i^	0.96	2.50	3.4386 (18)	165
C6—H6⋯O1^ii^	0.96	2.46	3.1856 (16)	133

Symmetry codes: (i) 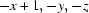; (ii) 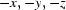.

**Fig. 1.**
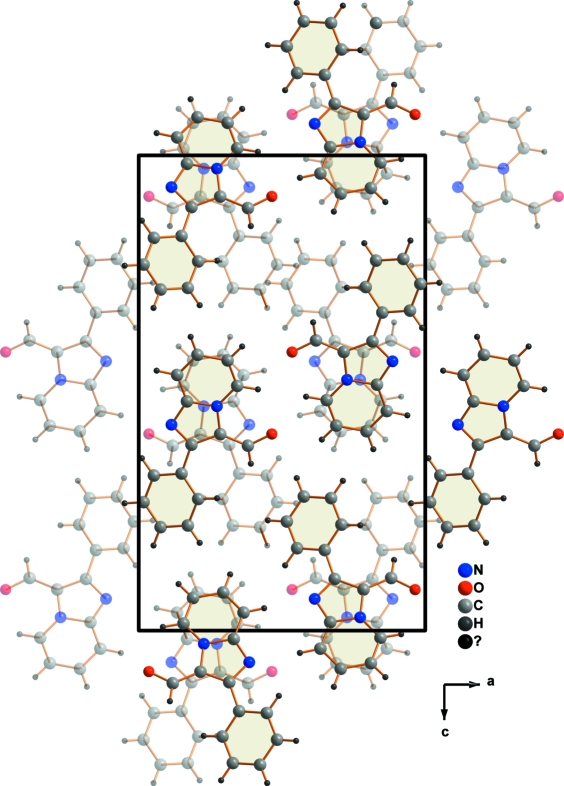
Packing of of Imidazo[1,2-a]pyridine-2-phenyl-3-carbaldehyde viewed along the b axis. Hydrogen bonds are not indicated.

**Fig. 2.**
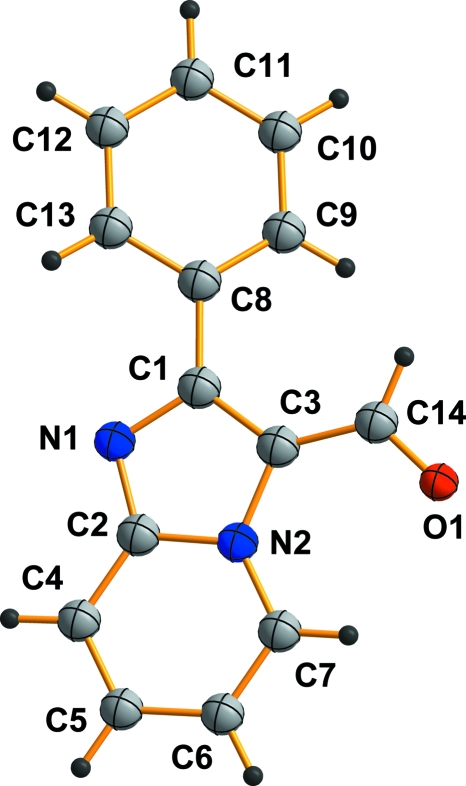
A molecule of the title compound, with 50% displacement ellispoids for non-H atoms.

**Fig. 3.**
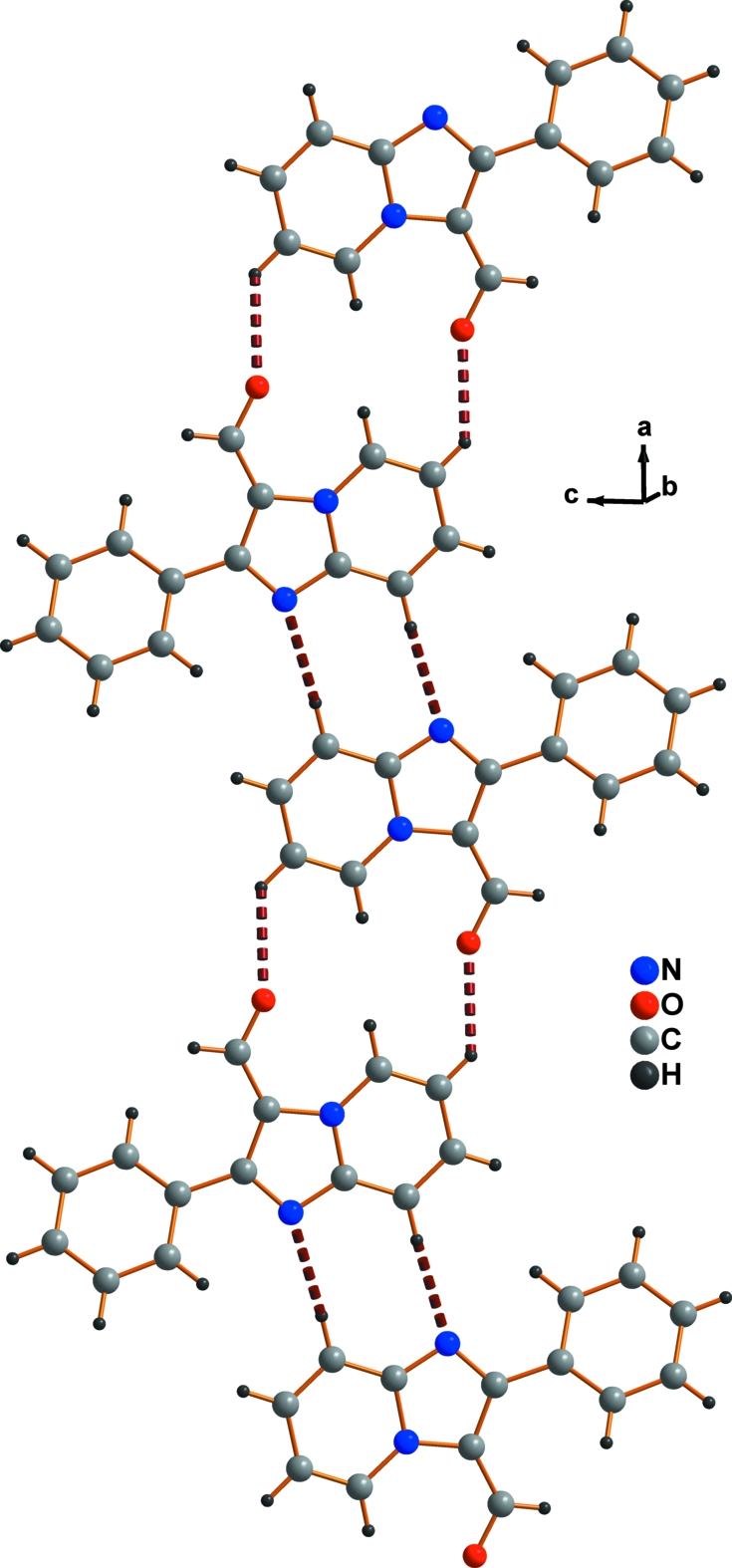
Part of a ribbon along the a axis showing intermolecular hydrogen bonds.

## supplementary crystallographic information

### Comment

Functionalized imidazo[1,2-*a*]pyridine and imidazo[1,2-*a*]pyrimidine systems are of great interest due to their biological activities (Anaflous *et al.*, 2008 and reference herein).

The structure of *N*-(2-phenylimidazo[1,2-*a*]pyridin-3-yl)acetamide(I) consists of isolated molecules which packing is shown in Fig. 1. The space conformation of the molecule (Fig. 2) is characterized by the dihedral angle of 28.61 (4) ° between the imidazo[1,2-*a*]pyridine and the phenyl rings. Weak C—H···N and C—H···O intermolecular hydrogen bonds (Table 1) connect the molecules into chains in the a direction (Fig. 3). The connection between ribbons along b (Fig. 1) is provided by π-π stacking interactions involving neighbouring pyridine rings with centroid-centroid distance 3.7187 (7) Å.

### Experimental

Imidazo[1,2-*a*]pyridine-2-phenyl-3-carbaldehyde was synthesized according to the method described by Vilsmeier-Haack (Meth-Cohn & Stanforth, 1991) : *i.e.* to 1.9 g (26 mmol) of DMF cooled at 273 K, containing 4 g (26 mmol) of phosphorus oxychloride (POCl_3_), was added portionwise to 10 mmole of 2-phenyl imidazo[1,2-*a*]pyridine. The mixture was heated at 373 K for 1 h. The solution was then neutralized at 273 K with Na_2_CO_3_ and extracted with Dichloromethane. The organic layer was dried over sodium sulfate and dichloromethane was removed under reduced pressure. The crude product was purified on silica gel column and imidazo[1,2-*a*]pyridine-2-phenyl-3-carbaldehyde was obtained in good yield (60%) as a white solid.

### Refinement

All the hydrogens (bonded to C atoms) were discernible in difference Fourier maps but according to standard procedures for organic compounds they were constrained to ideal positions (C-H: 0.96Å). Their isotropic atomic displacement parameters were evaluated as 1.2*U_eq_ of the parent atom.

### Crystal data

**Table:**

C_14_H_10_N_2_O	*F*_000_ = 928
*M**_r_* = 222.2	*D*_x_ = 1.406 Mg m^−^^3^
Orthorhombic, *P**b**c**a*	Mo *K*α radiation λ = 0.71069 Å
Hall symbol: -P 2ac 2ab	Cell parameters from 8746 reflections
*a* = 13.0640 (3) Å	θ = 2.7–26.5º
*b* = 7.4162 (2) Å	µ = 0.09 mm^−^^1^
*c* = 21.6698 (6) Å	*T* = 120 K
*V* = 2099.48 (9) Å^3^	Prism, colourless
*Z* = 8	0.57 × 0.40 × 0.24 mm

### Data collection

**Table:**

Oxford Diffraction Xcalibur2 diffractometer with Sapphire2 CCD detector	2196 independent reflections
Radiation source: X-ray tube	1305 reflections with *I* > 3σ(*I*)
Monochromator: graphite	*R*_int_ = 0.049
Detector resolution: 8.3438 pixels mm^-1^	θ_max_ = 26.6º
*T* = 120 K	θ_min_ = 3.1º
Rotation method data acquisition using ω scans	*h* = −16→16
Absorption correction: none	*k* = −9→9
25795 measured reflections	*l* = −27→27

### Refinement

**Table:**

Refinement on *F*^2^	H-atom parameters constrained
*R*[*F*^2^ > 2σ(*F*^2^)] = 0.031	Weighting scheme based on measured s.u.'s *w* = 1/[σ^2^(*I*) + 0.0016*I*^2^]
*wR*(*F*^2^) = 0.077	(Δ/σ)_max_ = 0.007
*S* = 1.04	Δρ_max_ = 0.15 e Å^−^^3^
2196 reflections	Δρ_min_ = −0.16 e Å^−^^3^
154 parameters	Extinction correction: none
36 constraints	

### Special details

**Table:**

Refinement. The refinement was carried out against all reflections. The conventional *R*-factor is always based on *F*. The goodness of fit as well as the weighted *R*-factor are based on *F* and *F*^2^ for refinement carried out on *F* and *F*^2^, respectively. The threshold expression is used only for calculating *R*-factors *etc*. and it is not relevant to the choice of reflections for refinement.All the H atoms were discernible in difference Fourier maps and could be refined to reasonable geometry. According to standard procedures for organic compounds the H atoms bonded to C atoms were constrained to ideal positions. The isotropic atomic displacement parameters of hydrogen atoms were evaluated as 1.2**U*_eq_ of the parent atom.The program used for refinement, Jana2006, uses the weighting scheme based on the experimental expectations, see _refine_ls_weighting_details, that does not force *S* to be one. Therefore the values of *S* are usually larger than the ones from the *SHELX* program.

### Fractional atomic coordinates and isotropic or equivalent isotropic displacement parameters (Å^2^)

**Table:**

	*x*	*y*	*z*	*U*_iso_*/*U*_eq_	
N1	0.38579 (8)	0.13099 (13)	0.06669 (5)	0.0227 (3)	
N2	0.22707 (8)	0.09004 (13)	0.02784 (5)	0.0204 (3)	
O1	0.03531 (7)	0.19646 (11)	0.08623 (4)	0.0297 (3)	
C1	0.31590 (10)	0.18666 (16)	0.10870 (6)	0.0208 (4)	
C2	0.33161 (10)	0.07295 (16)	0.01765 (6)	0.0207 (4)	
C3	0.21543 (10)	0.16659 (16)	0.08658 (6)	0.0204 (4)	
C4	0.36597 (11)	−0.00163 (16)	−0.03801 (6)	0.0235 (4)	
C5	0.29491 (10)	−0.05736 (17)	−0.08037 (7)	0.0243 (4)	
C6	0.18958 (10)	−0.03859 (17)	−0.06861 (6)	0.0247 (4)	
C7	0.15617 (10)	0.03543 (16)	−0.01503 (6)	0.0236 (4)	
C8	0.35054 (10)	0.25310 (16)	0.16940 (6)	0.0214 (4)	
C9	0.29235 (10)	0.23521 (17)	0.22302 (7)	0.0238 (4)	
C10	0.32855 (11)	0.30105 (17)	0.27874 (7)	0.0274 (5)	
C11	0.42372 (11)	0.38290 (17)	0.28223 (6)	0.0283 (5)	
C12	0.48307 (11)	0.39863 (17)	0.22959 (6)	0.0282 (4)	
C13	0.44708 (10)	0.33402 (16)	0.17344 (6)	0.0242 (4)	
C14	0.11885 (10)	0.22273 (16)	0.11073 (7)	0.0245 (4)	
H4	0.437543	−0.014444	−0.047038	0.0282*	
H5	0.318903	−0.109503	−0.118246	0.0292*	
H6	0.139117	−0.077351	−0.097954	0.0296*	
H7	0.084111	0.048834	−0.007645	0.0283*	
H9	0.226757	0.177012	0.221555	0.0286*	
H10	0.287357	0.289867	0.315233	0.0329*	
H11	0.448433	0.4285	0.320918	0.0339*	
H12	0.549259	0.454408	0.232038	0.0339*	
H13	0.489068	0.345338	0.137292	0.0291*	
H14	0.108688	0.287015	0.148695	0.0294*	

### Atomic displacement parameters (Å^2^)

**Table:**

	*U*^11^	*U*^22^	*U*^33^	*U*^12^	*U*^13^	*U*^23^
N1	0.0207 (6)	0.0225 (5)	0.0249 (6)	0.0004 (5)	0.0004 (5)	0.0017 (5)
N2	0.0196 (6)	0.0177 (5)	0.0239 (6)	−0.0005 (5)	−0.0001 (5)	0.0031 (5)
O1	0.0200 (6)	0.0315 (5)	0.0376 (6)	0.0000 (4)	−0.0002 (5)	0.0024 (5)
C1	0.0221 (7)	0.0149 (7)	0.0255 (8)	0.0007 (6)	0.0019 (6)	0.0052 (6)
C2	0.0192 (7)	0.0170 (6)	0.0259 (8)	−0.0002 (6)	0.0015 (6)	0.0051 (6)
C3	0.0211 (7)	0.0179 (6)	0.0223 (7)	−0.0008 (6)	0.0022 (6)	0.0026 (6)
C4	0.0219 (8)	0.0224 (7)	0.0263 (8)	−0.0005 (6)	0.0024 (6)	0.0027 (6)
C5	0.0281 (8)	0.0201 (7)	0.0248 (8)	−0.0015 (6)	0.0020 (6)	0.0031 (6)
C6	0.0245 (8)	0.0220 (7)	0.0275 (8)	−0.0044 (6)	−0.0037 (6)	0.0023 (6)
C7	0.0194 (8)	0.0225 (7)	0.0289 (8)	−0.0025 (6)	−0.0031 (6)	0.0043 (6)
C8	0.0228 (8)	0.0155 (6)	0.0260 (8)	0.0027 (6)	−0.0010 (6)	0.0032 (5)
C9	0.0216 (8)	0.0212 (7)	0.0288 (8)	−0.0003 (6)	−0.0001 (7)	0.0034 (6)
C10	0.0313 (8)	0.0267 (7)	0.0242 (8)	0.0049 (7)	0.0017 (7)	0.0034 (6)
C11	0.0330 (8)	0.0255 (7)	0.0263 (8)	0.0053 (7)	−0.0057 (7)	−0.0006 (6)
C12	0.0266 (8)	0.0242 (7)	0.0339 (8)	−0.0021 (6)	−0.0043 (7)	0.0013 (7)
C13	0.0243 (8)	0.0222 (7)	0.0263 (8)	0.0009 (6)	0.0007 (6)	0.0032 (6)
C14	0.0262 (8)	0.0201 (7)	0.0271 (8)	0.0008 (7)	0.0032 (7)	0.0041 (6)

### Geometric parameters (Å, °)

**Table:**

N1—C1	1.3537 (16)	C6—H6	0.9600
N1—C2	1.3475 (16)	C7—H7	0.9600
N2—C2	1.3893 (16)	C8—C9	1.3949 (19)
N2—C3	1.4020 (17)	C8—C13	1.3994 (18)
N2—C7	1.3731 (17)	C9—C10	1.386 (2)
O1—C14	1.2293 (16)	C9—H9	0.9600
C1—C3	1.4052 (18)	C10—C11	1.3857 (19)
C1—C8	1.4758 (18)	C10—H10	0.9600
C2—C4	1.4008 (18)	C11—C12	1.3840 (19)
C3—C14	1.4279 (18)	C11—H11	0.9600
C4—C5	1.3693 (19)	C12—C13	1.3897 (19)
C4—H4	0.9600	C12—H12	0.9600
C5—C6	1.4063 (18)	C13—H13	0.9600
C5—H5	0.9600	C14—H14	0.9600
C6—C7	1.3564 (19)		
			
C1—N1—C2	105.87 (10)	N2—C7—H7	121.20
C2—N2—C3	106.74 (10)	C6—C7—H7	120.02
C2—N2—C7	121.92 (11)	C1—C8—C9	122.93 (12)
C3—N2—C7	131.34 (11)	C1—C8—C13	118.38 (12)
N1—C1—C3	111.62 (11)	C9—C8—C13	118.67 (12)
N1—C1—C8	119.62 (11)	C8—C9—C10	120.43 (12)
C3—C1—C8	128.74 (12)	C8—C9—H9	120.11
N1—C2—N2	111.21 (11)	C10—C9—H9	119.46
N1—C2—C4	129.56 (12)	C9—C10—C11	120.53 (13)
N2—C2—C4	119.21 (11)	C9—C10—H10	119.74
N2—C3—C1	104.54 (11)	C11—C10—H10	119.73
N2—C3—C14	123.14 (12)	C10—C11—C12	119.65 (13)
C1—C3—C14	132.02 (12)	C10—C11—H11	120.24
C2—C4—C5	118.63 (12)	C12—C11—H11	120.11
C2—C4—H4	121.78	C11—C12—C13	120.19 (12)
C5—C4—H4	119.59	C11—C12—H12	119.70
C4—C5—C6	120.80 (13)	C13—C12—H12	120.11
C4—C5—H5	118.26	C8—C13—C12	120.50 (12)
C6—C5—H5	120.94	C8—C13—H13	120.09
C5—C6—C7	120.66 (12)	C12—C13—H13	119.40
C5—C6—H6	121.49	O1—C14—C3	125.44 (13)
C7—C6—H6	117.85	O1—C14—H14	109.03
N2—C7—C6	118.78 (12)	C3—C14—H14	125.53

### Hydrogen-bond geometry (Å, °)

**Table:**

*D*—H···*A*	*D*—H	H···*A*	*D*···*A*	*D*—H···*A*
C4—H4···N1^i^	0.96	2.50	3.4386 (18)	165
C6—H6···O1^ii^	0.96	2.46	3.1856 (16)	133

Symmetry codes: (i) −*x*+1, −*y*, −*z*; (ii) −*x*, −*y*, −*z*.

## References

[bb1] Anaflous, A., Albay, H., Benchat, N., El Bali, B., Dusek, M. & Fejfarova, K. (2008). *Acta Cryst.* E**64**, o926. 10.1107/S1600536808011501 PMC2961311 21202407

[bb2] Brandenburg, K. & Putz, H. (2005). *DIAMOND* Crystal Impact GbR, Postfach 1251, D-53002 Bonn, Germany.

[bb3] Burla, M. C., Camalli, M., Carrozzini, B., Cascarano, G. L., Giacovazzo, C., Polidori, G. & Spagna, R. (2003). *J. Appl. Cryst.* **36**, 1103.

[bb4] Meth-Cohn, O. & Stanforth, S. P. (1991). *Comp. Org. Synth.* **2**, 777–794.

[bb5] Oxford Diffraction (2006). *CrysAlis CCD* and *CrysAlis RED* Oxford Diffraction Ltd, Abingdon, Oxfordshire, England.

[bb6] Petříček, V., Dušek, M. & Palatinus, L. (2000). *JANA2000* Institute of Physics, Prague, Czech Republic.

